# Supplementary material for: The effects of a high-flavonoid corn cultivar on the gastrointestinal tract microbiota in chickens undergoing necrotic enteritis
Source: PLoS One. 2024 Sep 17;19(9):e0307333. doi: 10.1371/journal.pone.0307333 (PMC11407631; doi:10.1371/journal.pone.0307333)
Supplement: S1 Table — ASV = amplicon sequence variant; Feed = Feed A (commercial corn line-based diet) or Feed B (PennHFD1-based diet); Infection = Co-infection with E. maxima and C. perfringens or control (not infected). (DOCX) [file pone.0307333.s001.docx]

**SUPPLEMENTARY MATERIAL**

**The effects of a high-flavonoid corn cultivar on the gastrointestinal tract microbiota in chickens undergoing necrotic enteritis**

**S1 Table (Supplementary).** Effect of the main effects (feed and infection) and interactions on alpha diversity indices in the jejunal (JLC and JM) and ileal samples (ILC and IM) collected from 21 day-old chickens coinfected with E. maxima and C. perfringens.

| **Kruskal-Wallis *P-*values** | | | | |
| --- | --- | --- | --- | --- |
|  | **JLC** | **JM** | **ILC** | **IM** |
| **ASV** |  |  |  |  |
| Feed | 0.099 | 0.477 | 0.335 | 0.593 |
| Infection | 0.269 | 0.496 | 0.005 | 0.013 |
| Feed x Infection | 0.328 | 0.819 | 0.039 | 0.045 |
| **Shannon diversity index** |  |  |  |  |
| Feed | 0.533 | 0.286 | 0.852 | 0.888 |
| Infection | 0.507 | 0.891 | 0.172 | 0.035 |
| Feed x Infection | 0.876 | 0.707 | 0.443 | 0.038 |
| **Richness** |  |  |  |  |
| Feed | 0.075 | 0.328 | 0.535 | 0.255 |
| Infection | 0.122 | 0.618 | 0.576 | 0.004 |
| Feed x Infection | 0.200 | 0.289 | 0.621 | 0.005 |
| **Evenness index** |  |  |  |  |
| Feed | 0.374 | 0.248 | 0.901 | 0.886 |
| Infection | 0.564 | 0.389 | 0.950 | 0.059 |
| Feed x Infection | 0.636 | 0.309 | 0.960 | 0.143 |

**Legend:** ASV = amplicon sequence variant; Feed = Feed A (commercial corn line-based diet) or Feed B (PennHFD1-based diet); Infection = Co-infection with *E. maxima* and *C. perfringens* or control (not infected).
