# Supplementary material for: The effects of a high-flavonoid corn cultivar on the gastrointestinal tract microbiota in chickens undergoing necrotic enteritis
Source: PLoS One. 2024 Sep 17;19(9):e0307333. doi: 10.1371/journal.pone.0307333 (PMC11407631; doi:10.1371/journal.pone.0307333)
Supplement: S2 Table — Mean relative abundance (%) of taxonomic groups identified as relatively abundant at the genus and species level in jejunal (JLC and JM) and ileal (ILC and IM) samples collected from infected (co-infection with E. maxima and C. perfringens) and control (non-infected) chickens fed a commercial corn-based diet (A) or a high-flavonoid corn-based diet (B). CTL A (Non-infected chickens fed a commercial corn-based diet); CTL B (Non-infected chickens fed a PennHFD1-based diet); INF A (Chickens co-infected with E. maxima and C. perfringens fed a commercial corn-based diet); INF B (Chickens co-infected with E. maxima and C. perfringens fed a PennHFD1-based diet). * Statistical difference (ANOVA, P ≤ 0.05). (DOCX) [file pone.0307333.s002.docx]

**SUPPLEMENTARY MATERIAL**

**The effects of a high-flavonoid corn cultivar on the gastrointestinal tract microbiota in chickens undergoing necrotic enteritis**

**S2 Table (Supplementary).** Mean relative abundance (%) of taxonomic groups identified as relatively abundant at the genus and species level in jejunal (JLC and JM) and ileal (ILC and IM) samples collected from infected (co-infection with E. maxima and C. perfringens) and control (non-infected) chickens fed a commercial corn-based diet (A) or a high-flavonoid corn-based diet (B).

|  | **Treatment** | | | |
| --- | --- | --- | --- | --- |
| **Taxonomy** | CTL A | CTL B | INF A | INF B |
| Jejunal luminal content |  |  |  |  |
| **Genus** |  |  |  |  |
| *Clostridium* | 2.40 | 0.84 | 18.76 | 3.32 |
| *Lactobacillus* | 70.88 | 66.75 | 74.22 | 80.92 |
| LAR | 14.56 | 13.04 | 3.08 | 5.69 |
| UNCL | 12.15 | 19.35 | 3.92 | 10.05 |
| **Species** |  |  |  |  |
| *C. perfringens* | 2.40 | 0.79 | 18.76 | 3.26 |
| *L. reuteri* | 3.70 | 4.10 | 5.63 | 5.98 |
| *L. salivarius* | 5.17 | 5.96 | 4.92 | 5.69 |
| LAR | 6.62 | 5.20 | 3.35 | 4.31 |
| UNCL | 82.09 | 83.93 | 67.30 | 80.73 |
| Jejunal mucosa |  |  |  |  |
| **Genus** |  |  |  |  |
| *Bacteroides* | 10.14 | 5.43 | 6.92 | 3.44 |
| *Clostridium* | 1.95 | 0.04 | 12.27 | 0.42 |
| *Escherichia** | 0 | 0.15 | 2.39 | 0.65 |
| *Lactobacillus* | 32.18 | 41.19 | 49.47 | 69.48 |
| LAR* | 23.52 | 17.16 | 10.44 | 9.49 |
| UNCL* | 32.19 | 36.00 | 18.48 | 16.50 |
| **Species** |  |  |  |  |
| *B. fragilis* | 9.31 | 5.01 | 6.56 | 2.60 |
| *C. perfringens* | 1.95 | 0 | 12.27 | 0.40 |
| *E. coli** | 0 | 0.15 | 2.39 | 0.65 |
| *L. reuteri** | 2.40 | 2.68 | 4.83 | 12.47 |
| LAR | 6.02 | 5.47 | 5.53 | 5.09 |
| UNCL | 80.30 | 86.66 | 68.40 | 78.76 |
| Ileal luminal content  **Genus** |  |  |  |  |
| *Candidatus Arthromitus* | 14.15 | 0.40 | 0 | 0 |
| *Clostridium* | 1.68 | 0.17 | 16.55 | 15.33 |
| *Enterococcus** | 3.23 | 8.58 | 1.58 | 0.35 |
| *Escherichia* | 0.10 | 1.13 | 3.42 | 12.63 |
| SMB53* | 2.91 | 25.23 | 0.07 | 0.14 |
| *Lactobacillus* | 74.26 | 53.87 | 77.80 | 70.37 |
| LAR | 0.22 | 0.20 | 0.04 | 0.16 |
| UNCL | 3.42 | 10.40 | 0.50 | 0.99 |
| **Species** |  |  |  |  |
| *C. perfringens* | 1.68 | 0.03 | 16.55 | 15.33 |
| *E. cecorum** | 3.22 | 7.92 | 1.58 | 0.35 |
| *E. coli* | 0.10 | 1.13 | 3.42 | 12.63 |
| *L. salivarius* | 2.77 | 8.41 | 4.33 | 4.96 |
| LAR | 2.68 | 2.30 | 2.30 | 3.52 |
| UNCL | 89.53 | 80.18 | 71.79 | 63.17 |
| Ileal mucosa |  |  |  |  |
| **Genus** |  |  |  |  |
| *Clostridium* | 2.55 | 1.04 | 26.72 | 20.79 |
| *Enterococcus* | 1.79 | 2.99 | 2.45 | 0.45 |
| *Escherichia* | 1.27 | 0.57 | 3.98 | 7.12 |
| *Lactobacillus* | 28.27 | 38.30 | 62.37 | 56.22 |
| LAR* | 42.01 | 41.83 | 3.08 | 7.85 |
| UNCL* | 24.08 | 15.23 | 1.36 | 7.55 |
| **Species** |  |  |  |  |
| *B. fragilis** | 9.10 | 3.64 | 1.20 | 1.57 |
| *C. perfringens* | 2.53 | 0.966 | 26.72 | 20.79 |
| *E. cecorum* | 1.77 | 2.60 | 2.45 | 0.44 |
| *E. coli* | 1.27 | 0.57 | 3.98 | 7.12 |
| *L. reuteri** | 1.43 | 1.55 | 1.56 | 4.56 |
| *L. salivarius** | 1.88 | 4.52 | 1.41 | 1.44 |
| LAR* | 4.06 | 5.58 | 0.86 | 1.29 |
| UNCL | 77.93 | 80.52 | 61.78 | 62.75 |

**Legend:** CTL A (Non-infected chickens fed a commercial corn-based diet); CTL B (Non-infected chickens fed a PennHFD1-based diet); INF A (Chickens co-infected with *E. maxima* and *C. perfringens* fed a commercial corn-based diet); INF B (Chickens co-infected with *E. maxima* and *C. perfringens* fed a PennHFD1-based diet).

* Statistical difference (ANOVA, *P* ≤ 0.05).
