# Supplementary material for: The effects of a high-flavonoid corn cultivar on the gastrointestinal tract microbiota in chickens undergoing necrotic enteritis
Source: PLoS One. 2024 Sep 17;19(9):e0307333. doi: 10.1371/journal.pone.0307333 (PMC11407631; doi:10.1371/journal.pone.0307333)
Supplement: Attachment — Submitted filename: Rebuttal_Plos_One_Buiatte_2.docx [file pone.0307333.s003.docx]

**Manuscript:** PONE-D-24-09821

Dear Dr. Kogut, ***PLOS ONE*** Academic Editor,

We are pleased to submit the revised draft of our manuscript “**The effects of a high-flavonoid corn cultivar on the gastrointestinal tract microbiota in chickens undergoing necrotic enteritis**” for consideration of publication. We are thankful for the opportunity to receive constructive feedback that helped improve our manuscript. We believe that we were able to address most of your concerns.

Please see below a point-by-point response to the reviewers’ comments. Our answers in this letter are highlighted in blue color, and the changes made to the manuscript are highlighted in yellow.

Sincerely,

**Alberto Gino Lorenzoni, DVM, MS, PhD.**

Associate Professor of Poultry Science and Avian Health

Department of Animal Science

The Pennsylvania State University

Corresponding Author

**Reviewer’s comments to the authors:**

**Reviewer #1**

The results are interesting but there is a need to expand the discussion besides what they were expecting regarding the microbiota modulation. I see an important limitation in the NE challenge model regarding the interpretation of "mechanism of action". Coccidia is also being affected by the flavonoids, and it is at least the 50% of the changes that they are introducing there!
The study is a based in a challenge model that uses infection with coccidia as a predisposing factor for the development of C. perfringens necrotic enteritis. Although this is a very common NE challenge model used in several studies, and also imposed to the industry as almost a gold standar, the inclusion of feed additives or diet to compare treatments against a non-challenged control must be carefully interpreted. Many of these additives (or diet components) can alter the infection of coccidia (either in the level as well as the cycle (i.e. shortening or extending) by direct interaction with the parasite or the physiology of the host. Particularly flavonoids are able to inhibit the invasion and replication of different species of coccidian and also alter epithelial physiology and immune responses of the gut. As C. perfringens challenge is synchronized at a fix time after coccidian delivery, if cycling is lightly altered, it can influence how C. perfringens induce lesions. I would suggest to include this discussion in the paper, limiting the expected outcomes of the results obtained.

Thanks for taking the time to thoroughly review our manuscript and give us feedback.

We agree with your comments. We expanded the discussion with supporting literature that considers the other factors that interplay in the effects observed in our study. (Page 21, lines 445-457).

In lines 481-483 the authors state that the results "indicate" that microbiota modulation is an important mechanism of action of high-flavonoid corn in the observed reduction of necrotic enteritis. However, although the results show a modulation of the microbiota, they are not strong enough, due to the experimental design and the co-infection challenge model used, to "indicate" that it is a mechanism of action. The results do show that there is a change in the microbiota and that these changes are associated with a decrease in the number (?) of animals with lesions compatible with necrotic enteritis (I understand that there would be no differences in the degree of lesion, the authors refer to a previous work that is supposed to continue in this one) but there is no direct relationship. These results could suggest a potential role of the microbiota in the observed effects. However, if flavonoids are altering for example intestinal transit, water homeostasis, mucus secretion or antimicrobial peptides secretion, then a microbiota modulation would be also observed. Please consider the potential changes in animal physiology as part of the mechanisms of action of the high-flavonoid corn on the observed microbiota changes.

We agree with your comment. We made changes to the text (Page 23, Lines 492-495; and Lines 497-502) and added supporting information (Page 21, lines 444-457) to explore the other factors that were not evaluated in this study.

**Reviewer #2:**
1. The number of replicates is very small to make any logical conclusions.

We refined our conclusion to account for pitfalls related to experimental design (Page 23, Lines 496-502).

2. provide the flavainoid content of the corn.

We added a sentence reporting the flavonoid content of both corn lines used in the study, and referenced previously reported data (Page 6, Lines 147-148).

**Reference:**

Buiatte V, Dominguez D, Lesko T, Jenkins M, Chopra S, Lorenzoni AG. Inclusion of high-flavonoid corn in the diet of broiler chickens as a potential approach for the control of necrotic enteritis. Poult Sci. 2022;101. doi:10.1016/j.psj.2022.101796

3. provide the basic nutritive value of the corn under study.

The complete wet chemistry analyses of the corn lines have been previously reported (Figure below). We added a sentence in the materials and methods to address your concern. (Page 6, Lines 148-149).

**Reference:**

Buiatte V, Dominguez D, Lesko T, Jenkins M, Chopra S, Lorenzoni AG. Inclusion of high-flavonoid corn in the diet of broiler chickens as a potential approach for the control of necrotic enteritis. Poult Sci. 2022;101. doi:10.1016/j.psj.2022.101796

Source: Buiatte et al. (2022), *Poultry Science.*
